# Biotin/sulfasalazine combination therapy alleviates acetic acid-induced ulcerative colitis in rats via modulation of S1PR1/NF-κB/IL-23/STAT3/COX-2 axis

**DOI:** 10.1038/s41598-025-09932-w

**Published:** 2025-07-25

**Authors:** Sahar A. Helmy, Mahmoud M. Samaha, Al Shaima G. Abd El Salam, Nesma A. Abd Elrazik

**Affiliations:** 1 Department of Biochemistry, Faculty of Pharmacy, Mansoura University, P. O. Box 35516, Mansoura, Egypt; 2 Department of Pharmacology and Toxicology, Faculty of Pharmacy, Mansoura University, P. O. Box 35516, Mansoura, Egypt

**Keywords:** Ulcerative colitis, Sulfasalazine, Biotin, S1PR1, STAT3, IL-23, Biochemistry, Ulcerative colitis

## Abstract

Ulcerative colitis (UC) is a chronic idiopathic mucosal inflammation of colon. The lack of effective remedies urges us to search for new remedies to effectively cure UC. The current study aims to explore the potential therapeutic effect of sulfasalazine (SLZ)/Biotin combination in ameliorating acetic acid (AA)-evoked UC in rats. SLZ (100 mg/kg), Biotin (6 mg/kg) and SLZ plus Biotin were administered orally for 8 days followed by injection of AA (2 mL, 3% v/v) intra-rectally to induce UC on the 8^th^ day. SLZ/Biotin combination therapy attenuated AA-induced UC as proved by mitigation of pathological colonic abnormalities and decrease in disease activity index, colon mass index, colon weight/length ratio, LDH and CRP serum levels. This was associated with a considerable restoration of redox state in colon; MDA, NO and GSH contents. Furthermore, SLZ/Biotin combination therapy reduced colonic inflammation as confirmed by the remarkable decrement of S1P, S1PR1, IL-23, STAT3, and P-STAT3 colonic levels along with downregulation of colonic COX-2 and NF-κB protein expressions. Biotin as add-on therapy to SLZ markedly alleviates AA-induced UC via modulating S1P/S1PR1/NF-kB/ IL-23/STAT3 inflammatory signaling pathway with subsequent inhibition of COX-2.

## Introduction

Ulcerative colitis (UC) is an idiopathic chronic inflammation of the colon in which a deep-rooted inflammation is developed in the innermost colonic tissue and rectum. It is considered the most widespread form of inflammatory bowel disease (IBD) around the world^1–3^. In 2023, UC’s prevalence was estimated to be five million cases worldwide, and its incidence is rising globally^4^. Unfortunately, UC is a lifelong disorder affecting both mental and physical health and has no exact cure^2,3^. Patients with UC are at a two to threefold increased risk of developing colorectal cancer than the general population^5^.

Recent studies shed the light on sphingosine-1 phosphate receptor 1 (S1PR1) modulating therapies in treatment of UC as a promising alternative to traditional drugs^6,7^. They are considered the newest class of small oral molecules approved by FDA for UC treatment. They act via targeting the interaction between S1PR1 and its ligand, which in turn control lymphocyte egress from lymph nodes and spleen into systemic circulation, and thereby suppressing inflammation in IBD^7^. Moreover, activation of S1PR1 leads to persistent activation of signal transducer and activator of transcription 3 (STAT3) and nuclear factor kappa B (NF-κB) which are implicated in the development of chronic intestinal inflammation and colitis-associated colorectal cancer^8^. Also, stimulation of interleukin-23 (IL-23), a NF-κB downstream target, triggers STAT3 signaling that plays an important role in UC pathogenesis and is correlated with disease severity^9^. The evidence that S1PR1 amplifies inflammatory response has opened new avenues to explore S1PR1/ NF-κB/ IL-23/ STAT3 axis as encouraging therapeutic targets in UC.

Sulfasalazine (SLZ) is one of the most widespread drugs used for the management of UC^10^. It proved its efficacy as both treatment for inducing remission and maintenance treatment^4^. Despite the promising efficacy of SLZ, about 10 to 20% of UC patients undergo proctocolectomy for medically refractory disease. Thus, the combination therapy is regarded to be one of the keys to breaking through this therapeutic ceiling^4^.

Biotin is an essential micronutrient obtained from exogenous sources like egg yolk, vegetables, and liver^11^. Clinically, marginal or severe biotin deficiency is associated with immunodeficiency, dermatitis, conjunctivitis, and IBD^12–14^. Moreover, an aberrant rise in the levels of proinflammatory cytokines has been observed in biotin deficiency, supporting its inflammatory-modulating effect^15,16^. Thus, we were motivated to study the effect of biotin as add on therapy to SLZ in acetic acid (AA)-induced UC rat model. AA has been widely used to induce UC in various experimental studies^17–20^. AA-evoked UC model in rats is deemed to extremely mimic the human UC pathogenesis and histological features and open-up new areas to explore therapeutic intervention strategies^20^.

To the best of our knowledge, the ability of SLZ/Biotin combination in managing UC has not yet been studied. Given the importance of combination therapy in UC management, this study aims to investigate the potential therapeutic effect of SLZ/Biotin combination in ameliorating AA-evoked UC in rats and highlight the underlying mechanism implicated in its anti-ulcerative impact.

## Materials and methods

### Drugs and chemicals

SLZ bought as Colosalazine-EC® tablet (Arab Caps, Egypt). Biotin was obtained from Sigma-Aldrich (St. Louis, MO, USA). AA was purchased from CID Pharmaceutical Co. (Giza, Egypt). All other used chemicals were of the highest analytical grade and purity.

### Animals

A total of 40 male Sprague Dawley rats weighing 250 g ± 50 g were acquired from Mansoura University’s Medical Experimental Research Centre (MERC). For a duration of two weeks, the rats were housed in typical laboratory settings, which included a 12-h light/dark cycle, a regulated room temperature of 25 ± 1 °C, and unrestricted access to water and pellet food. The rats were starved for 24-h before the research ended. The Animal Care and Use Committee of Mansoura University in Egypt authorized all studies under the ethical code **MU-ACUC (PHARM.R.23.12.28)**, in compliance with the National Research Council publication "Guide for the Care and Use of Laboratory Animals" (8th ed., USA, 2011). The present study has adhered to the ARRIVE guidelines (https://arriveguidelines.org) with the purpose of ensuring high standards, reliability, and transparency in animal research.

### Induction of UC

To induce UC, rats were overnight fasted, anesthetized with thiopental sodium (20 mg/kg, I.P), and then 2 mL of diluted AA (3% v/v in 0.9% saline) was instilled slowly into rat’s colon using a polyurethane tube for enteral feeding (2 mm in diameter, 8 cm in length). To avoid AA leakage, rats were kept in a supine Trendelenburg position during the rectal instillation process and for two minutes afterward^21,22^.

### Experimental design and sample collection

Rats were randomly divided into 5 groups (8 rats each) as follows:

- Control group: For 8 days, rats were given 0.5% carboxymethyl cellulose (CMC) orally as a vehicle. On the 8^th^ day, animals underwent the same intra-rectal technique outlined in Section “Induction of UC” to receive 2 mL of normal saline in place of AA.
- AA-induced UC group: Rats received 0.5% carboxymethyl cellulose (CMC) for 8 days as a vehicle orally and on the 8^th^ day, rats intra-rectally received 2 mL of AA by the same procedure described in Section “Induction of UC”.
- AA + SLZ group: Rats were given SLZ (100 mg/kg) orally for 8 days, and on the 8th day, AA was given intra-rectally^21^.
- AA + Biotin group: Rats were given biotin (6 mg/kg) orally for 8 days, and on the 8^th^ day, AA was given intra-rectally^23^.
- AA + SLZ + Biotin group: Rats were given SLZ (100 mg/kg) and biotin (6 mg/kg) orally for 8 days, and on the 8^th^ day, AA was given intra-rectally.

**Fig. 1:**
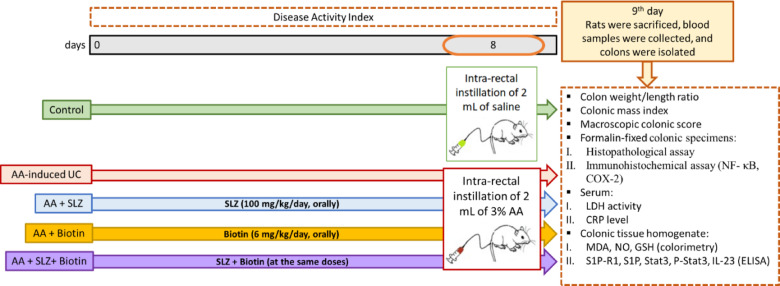
Experimental design and sample collection. AA: acetic acid; COX-2: cyclooxygenase-2; CRP: C-reactive protein; GSH: glutathione; IL-23: interleukin-23; LDH: lactate dehydrogenase; MDA: malondialdehyde; NF-κB: nuclear factor kappa B; NO: nitric oxide; S1PR1: sphingosine-1 phosphate receptor 1; SLZ: sulfasalazine; STAT3: signal transducer and activator of transcription 3; UC: ulcerative colitis.

On 9th day, rats were weighed and anaesthetized with I.P injection of 40 mg/kg thiopental sodium, samples of blood were obtained via retro-orbital puncture, to separate sera, and preserved at − 80 °C for biochemical investigations. Then, rats were euthanized by cervical dislocation, distal colon samples (6–8 cm in length) were isolated, washed with saline, weighed, and imaged. Part of the distal colon samples (1 cm in length) was instantly fixed with 10% neutral buffered formalin and embedded in paraffin for histopathological and immunohistochemical examination. The other part was stored at − 80 °C for biochemical analyses. There were three repeats for each experiment. A schematic diagram of experimental procedures is presented in Fig. 1.

### Disease activity index (DAI)

The disease activity index (DAI) was used to quantify the clinical evolution of UC^24^.The changes in weight, stool consistency and presence of gross bleeding or occult blood in feces were daily scored from 0 to 4 for each rat, as shown in Table 1.

**Table 1:** Disease activity index (DAI) scoring system.

Scores	Body weight loss (%)	Diarrhea	Rectal bleeding
0	None	Normal	Normal
1	1–5%	Soft stool but still formed	Positive hemoccult
2	6–10%	Very soft stool	Blood traces in stool visible
3	11–20%	Mild diarrhea	Mild bleeding
4	> 20%	Severe diarrhea	Severe bleeding

DAI = (body weight loss score + stool consistency score + bloody stool score)/3.

### Colon mass index

Colon mass index can reflect the degree of intestinal edema and the intensity of colonic inflammation. It was calculated by the following formula: colon mass index = colon weight (g)/total body weight (g) × 100.

### Colon weight/length ratio

Each rat’s weight/length ratio was estimated after the weight and length of the separated colons were assessed.

### Macroscopic colonic score

The macroscopic colonic score was assessed as previously described by Mei et al.^25^. Colons were sliced, opened longitudinally, and cleaned with 0.9% (w/v) normal saline after the animals were scarified. Table 2 represents the criteria used to evaluate macroscopic damage based on clinical symptoms on an arbitrary scale from 0 to 4.

**Table 2:** Macroscopic colonic scoring system.

Scores	Macroscopic colonic changes
0	None
1	mucosal erythema alone
2	mild mucosal edema, modest bleeding ulcers or erosions
3	moderate edema, slight bleeding ulcers or erosions
4	severe ulceration, edema, and tissue necrosis

### Histopathological analysis

Colon specimens were preserved in 10% neutral buffered formalin for 24 h, washed with tap water, dehydrated, cleared in xylene, routinely prepared to obtain 5–6 μm-thick paraffin-embedded sections and stained using hematoxylin and eosin (H&E)^26^. Slides were photographed using MVV5000CL digital eyepiece installed on MEIJI MX5200L microscope and Future WinJoe software, using a 10X objective. Histopathological scoring system evaluating major structural alterations in the colon was performed as previously described by Deshmukh et al. (Table 3)^27^.

**Table 3:** Colonic tissue inflammation scoring system.

Scores	Histopathological colonic changes
0	None
1	mucosal erythema alone
2	mild mucosal edema, slight bleeding, or slight erosion
3	moderate edema, bleeding ulcers, or erosions
4	severe edema, ulceration, erosions, and necrosis

### Immunohistochemical analysis

Colonic sections were mounted on slides, deparaffinized in xylene and rehydrated in ethanol solutions of different grades^28,29^. Antigen retrieval and blocking of the nonspecific background were conducted as previously described by Abu-Elala et al.^30^. Slides were incubated at 4 °C overnight with primary antibodies for NF-κB (ab32360) and cyclooxygenase-2 (COX-2) (ab179800) supplied by Abcam, Inc (Cambridge, UK). Slides were washed three times with Tris-buffered saline and incubated with a species-matched secondary antibody that was added for 1 h at room temperature. 3,3-diaminobenzidine (DAB) was used as a chromogen to visualize immune-stained cells. Colonic sections were stained with Mayer hematoxylin and mounted. NF-κB and COX-2 protein expressions were measured and analyzed in stained areas in 5 random fields per group via image analyzer software (Fiji ImageJ, version 1.51r; NIH, Maryland, USA).

### Preparation of colonic tissue homogenate

Colonic tissues were homogenized in 10% w/v phosphate-buffered saline and then centrifuged at 8000 rpm, 4 °C for 20 min using Sigma D-37520 centrifuge (Sigma Laborzentrifugen GmbH, Germany). The supernatant separated to be used for further biochemical analyses.

### Biochemical assays

Serum lactate dehydrogenase (LDH) activity and C-reactive protein (CRP) level were measured using commercially available spectrophotometric kits (Human diagnostics, Wiesbaden, Germany) and (spinreact, Barcelona, Spain), respectively, in accordance with manufacturer’s instructions. Colonic tissue homogenates were assessed for malondialdehyde (MDA), nitric oxide (NO), and glutathione (GSH) spectrophotometrically using commercially available kits provided by Biodiagnostic (Giza, Egypt) for each parameter following the provided directives. Also, S1PR1, sphingosine-1 phosphate (S1P), STAT3, P-STAT3, IL-23 were determined in colonic tissue homogenates using rat ELISA kits provided by Assay Genie (#RTEB0963, Duke St., London), Abbexa Ltd. (#abx585002, Cambridge, UK), MyBioSource (#MBS3809235, San Diego, California, USA), AFG Scientific (#EK72164, Northbrook, USA) and MyBioSource (#MBS704680, San Diego, California, USA), respectively. Sample absorbance was assessed using BioTek ELISA Reader (Winooski, USA).

### Statistical analysis

Data were expressed as means ± SEM. Multiple group comparisons were carried out using one-way analysis of variance (ANOVA), followed by Tukey’s post-hoc test for multiple comparisons, except that for disease activity index, macroscopic colonic score and histopathological score assessment, they were performed using Kruskal–Wallis test followed by Dunn’s multiple comparison post-hoc tests and data were expressed as median and interquartile range (IQR). Statistics and graphical presentations were created using GraphPad Prism version 9.0.0 (Graph Pad Software Inc., SanDiego, USA). Statistical significance was fixed at *p* ≤ 0.05.

## Results

### Effect of SLZ and biotin on AA-induced colonic histopathological changes

**Fig. 2:**
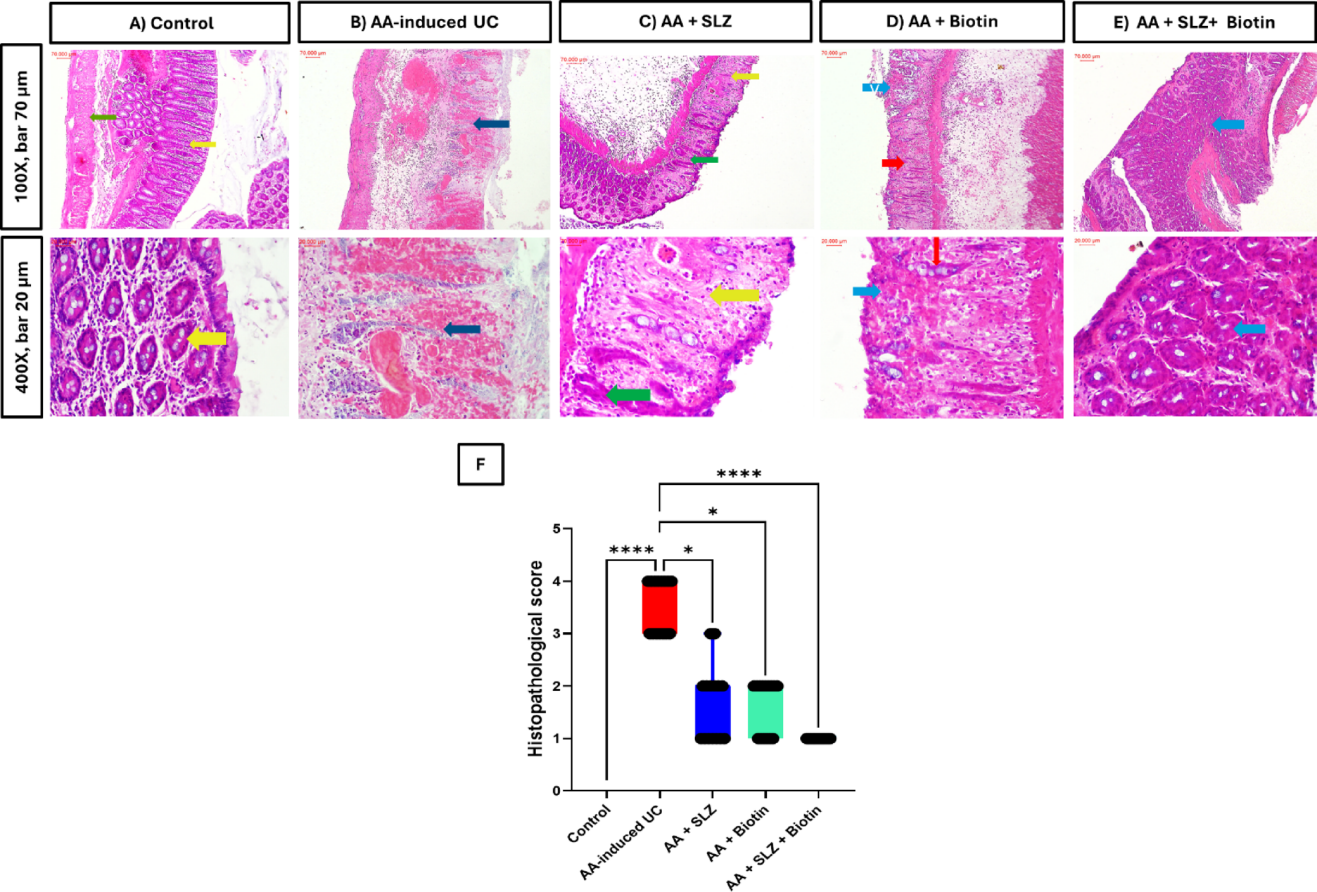
Effect of SLZ and biotin on colon histopathological changes induced by AA in rats Representative photomicrographs of H&E-stained colonic sections. (**A**) Control group: showed normal mucosa with uniform colonic glands lined with mucin secreting epithelial cells (yellow arrows). (**B**) AA-induced UC group: showed total ulceration of colonic wall with loss of mucosal glands with extensive haemorrhage (blue arrows). (**C**) AA + SLZ group: showed focal ulceration seen as loss of colonic glands in part of mucosal lining (yellow arrows) and remaining intact colonic glands (green arrows). (**D**) AA + Biotin group: showed focal ulceration seen as loss of colonic glands in part of mucosal lining (blue arrows) and remaining intact colonic glands (red arrows). (**E**) AA + SLZ + Biotin group: showed normal colonic mucosa (blue arrows). Upper panel (100X, bar 70 μm) and lower panel (400X, bar 20 μm). (**F**) Colonic histopathological scores, values are expressed as median and interquartile range (IQR). (n = 6). *p < 0.05, ****p < 0.0001. AA, acetic acid; SLZ, sulfasalazine; UC, ulcerative colitis.

The H&E-stained colonic sections of control group showed normal mucosa with uniform colonic glands lined with mucin secreting epithelial cells. However, the AA-induced UC group revealed total ulceration of colonic wall with loss of mucosal glands and extensive haemorrhage. Both SLZ and biotin groups revealed moderate and minor inflammation in mucosa and submucosa, respectively. While SLZ/Biotin combination group attenuated colonic histopathological changes induced by AA as the colonic sections showed an almost preserved mucosa with healthy submucosa (Fig. 2).

### Effect of SLZ and biotin on DAI and macroscopic colonic scores

**Fig. 3:**
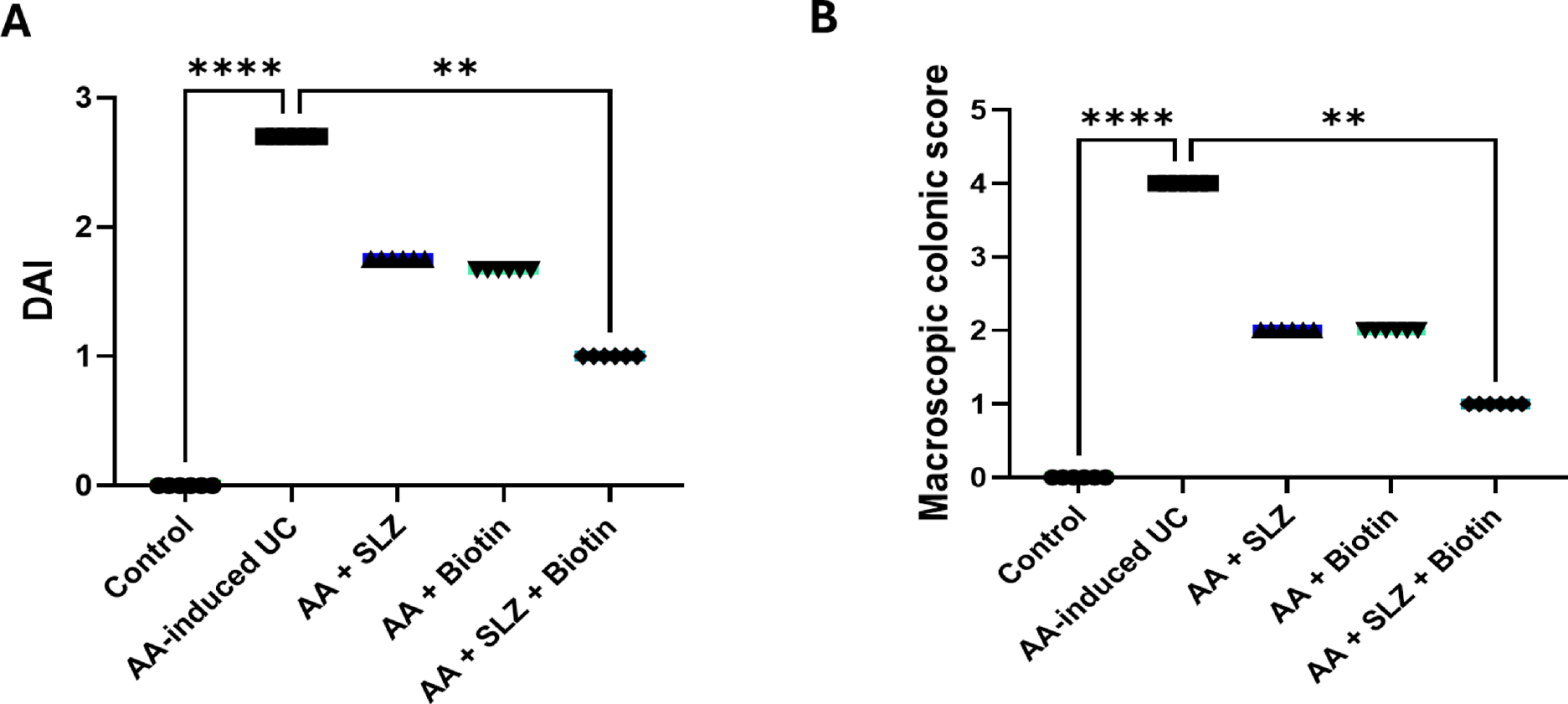
Effect of SLZ and biotin on DAI and macroscopic colonic scores in AA-induced UC in rats. (**A**) DAI score; (**B**) macroscopic colonic score. Values are expressed as median and interquartile range (IQR) (n = 6). **p < 0.01, ****p < 0.0001. AA, acetic acid; DAI, disease activity index; SLZ, sulfasalazine; UC, ulcerative colitis.

The AA-induced UC rats revealed marked elevation in DAI when compared to control rats. Rats treated with SLZ or biotin showed non-significant lowering in DAI when compared to AA-induced UC rats, while the rats treated with SLZ/Biotin combination therapy showed significant lowering in DAI by 2.7-folds when compared to AA-induced UC rats (Fig. 3A).

As shown in (Fig. 3B), macroscopic exploration of colons from AA-induced UC rats revealed apparent mucosal erythema, necrosis, ulceration, and inflammation compared to colons of control rats which showed normal mucosal architecture. On the contrary, the administration of SLZ/Biotin combination therapy alleviated mucosal alterations remarked in colons of AA-induced UC rats. In addition, treatment with SLZ or biotin alone revealed non-significant attenuation of macroscopic colonic changes.

### Effect SLZ and biotin on colon mass index and colon weight/length ratio changes

The AA-induced UC group revealed marked elevation in colon mass index and colon weight/length ratio by 1.5- and 1.5-folds, respectively when compared with control group. The SLZ and biotin-treated groups showed non-significant lowering in colon mass index and colon weight/length ratio when compared to AA-induced UC group. On the other hand, SLZ/Biotin combination therapy revealed a significant lowering in colon mass index and colon weight/length ratio by 1.4- and 1.5-folds, respectively as compared to AA-induced UC group **(**Table 4**).**

**Table 4:** Effect of SLZ and biotin on colon mass index and colon weight/length ratio changes induced by AA in rats.

Group	Colon mass index	Colon weight/length ratio
Control	1.7 ± 0.1	98.8 ± 0.8
AA-induced UC	2.6 ± 0.2^a^	152.5 ± 11.0^a^
AA + SLZ	2.4 ± 0.2	133.1 ± 10.0
AA + Biotin	2.1 ± 0.1	131.9 ± 9.9
AA + SLZ + Biotin	1.9 ± 0.1^b^	104.4 ± 9.5^b^

Data are expressed as mean ± SEM (n = 6).

Statistically significant: ^a^*p* < 0.05, compared with control group, ^b^*p* < 0.05, compared with AA-induced UC group.

### Effect of SLZ and biotin on serum LDH activity and CRP level

**Fig. 4:**
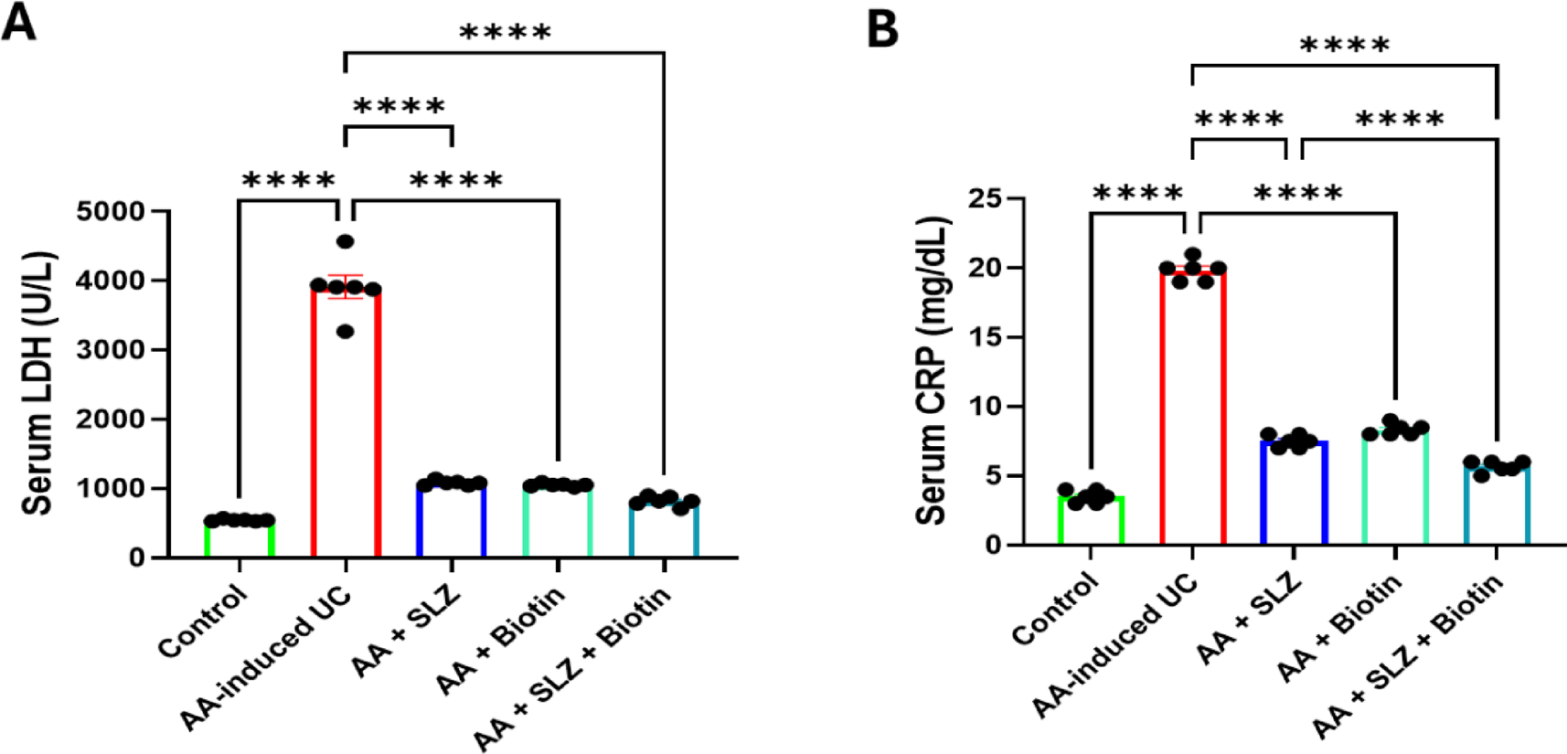
Effect of SLZ and biotin on serum LDH activity and CRP level in AA-induced UC in rats. (**A**) Serum LDH activity. (**B**) Serum CRP level. Values are expressed as mean ± SEM (n = 6). ****p < 0.0001. AA, acetic acid; CRP, C-reactive protein; LDH, lactate dehydrogenase; SLZ, sulfasalazine; UC, ulcerative colitis.

As revealed in Fig. 4, intra-rectal administration of AA accompanied with marked increment in serum LDH activity and CRP level by 7.2- and 5.7-folds, respectively, compared with control rats. Rats treated with SLZ or biotin alone showed significant decrease in serum LDH activity and CRP level compared with AA-induced UC rats. Furthermore, rats treated with SLZ/Biotin combination therapy showed more marked effect as it lowered serum LDH activity and CRP level by 4.8- and 3.5-folds, respectively, compared with AA-induced UC rats. Moreover, SLZ/Biotin combination group revealed significant lowering in serum CRP level as compared with SLZ-treated group.

### Effect of SLZ and biotin on redox state in colon tissues

**Fig. 5:**
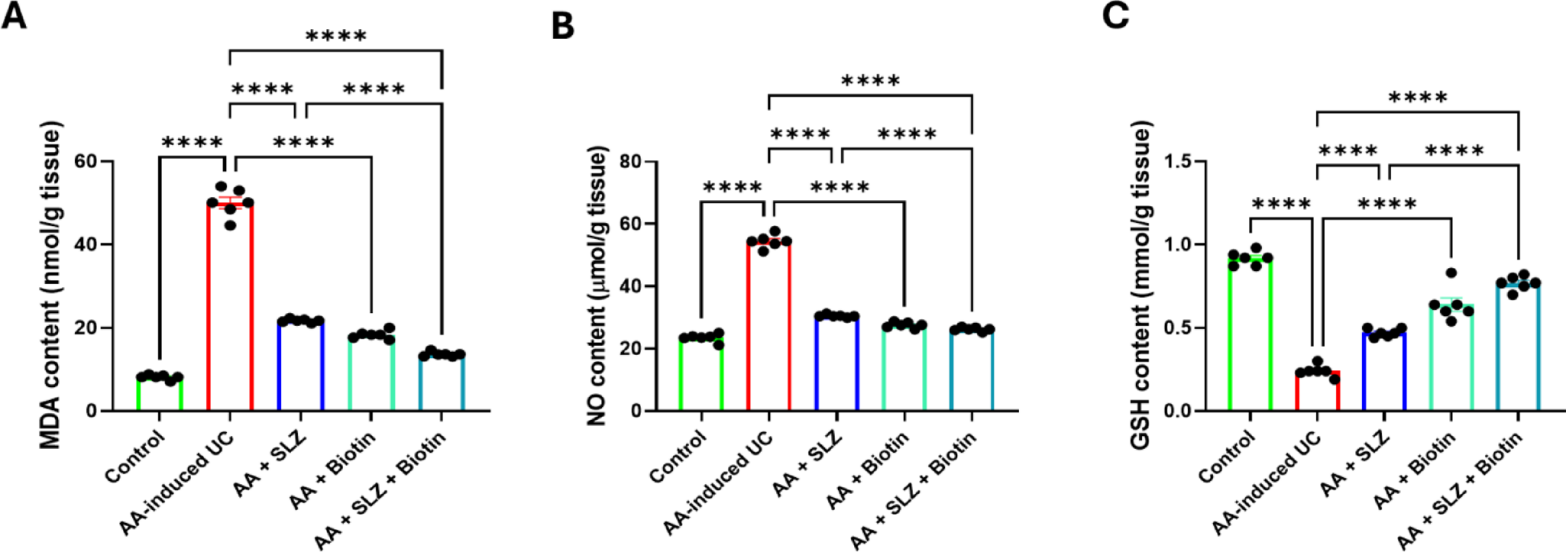
Effect of SLZ and biotin on redox state in colon in AA-induced UC in rats. (**A**) Colonic MDA content. (**B**) Colonic NO content; (**C**) Colonic GSH content. Values are expressed as mean ± SEM. (n = 6). ****p < 0.0001. AA, acetic acid; GSH, glutathione; MDA, malondialdehyde; NO, nitric oxide; SLZ, sulfasalazine; UC, ulcerative colitis.

The AA-induced UC group showed a notable rise in colonic MDA and NO contents along with marked lowering in colonic GSH content, compared to control group. All treatment groups revealed marked attenuation of AA-induced colonic oxidative stress as confirmed by significant elevation in colonic GSH content along with significant decrease in colonic MDA and NO content. Moreover, the antioxidant activity of SLZ/Biotin combination therapy superimposed SLZ alone as it markedly decreased colonic MDA content along with increased colonic GSH content by 1.6- and 1.6-folds, respectively (Fig. 5).

### Effect of SLZ and biotin on S1P and S1PR1 colonic levels

**Fig. 6:**
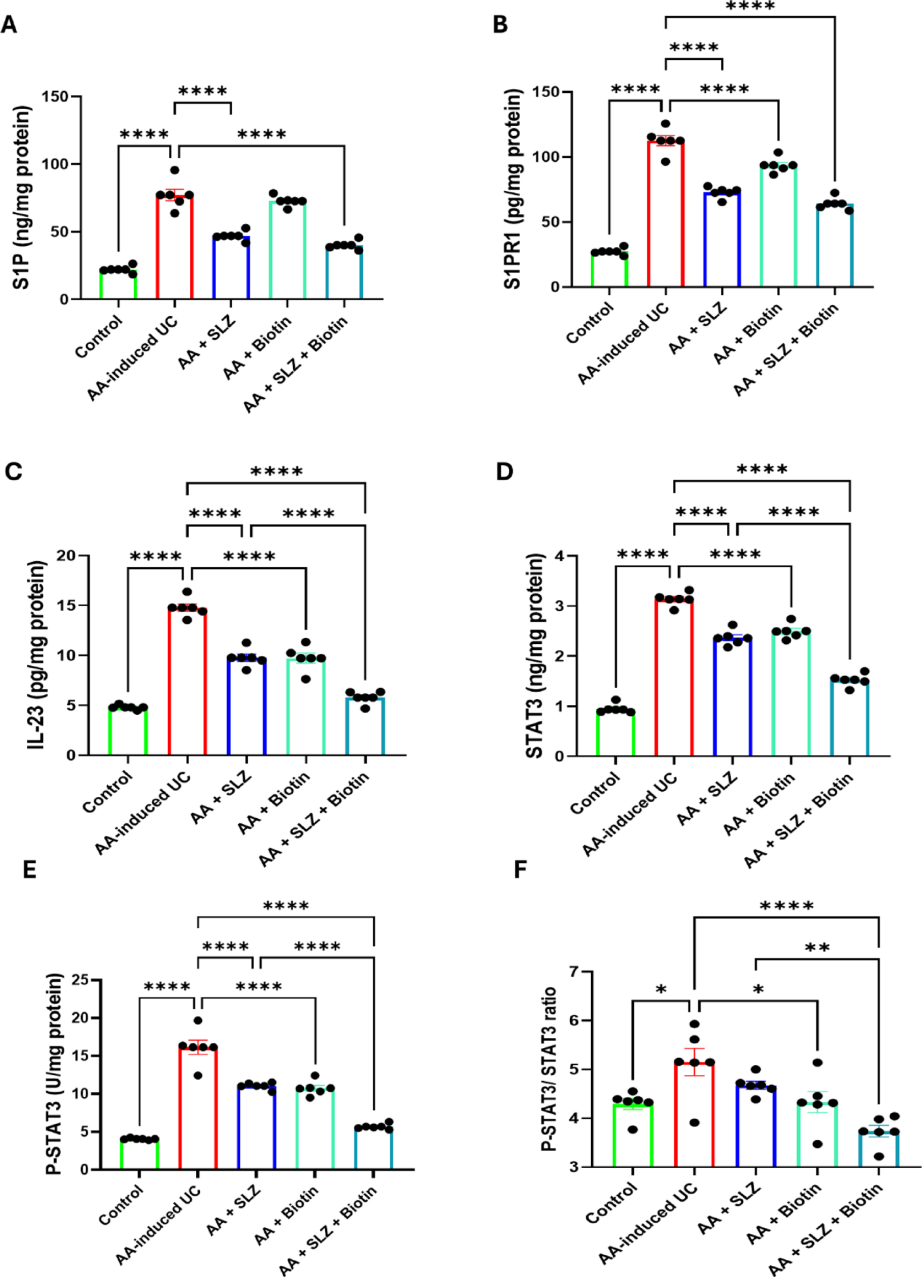
Effect of SLZ and biotin on S1P, S1PR1, IL-23, STAT3 and P-STAT3 colonic levels in AA-induced UC in rats. (**A**) Colonic S1P level. (**B**) Colonic S1PR1 level. (**C**) Colonic IL-23 level. (**D**) Colonic STAT3 level. (**E**) Colonic P-STAT3 level. (**F**) Colonic P-STAT3/STAT3 ratio. Values are expressed as mean ± SEM. (n = 6). *p < 0.05, **p < 0.01, ****p < 0.0001. AA, acetic acid; IL-23, interleukin-23; S1P, sphingosine-1 phosphate; S1PR1, sphingosine-1 phosphate receptor 1; SLZ, sulfasalazine; STAT3, signal transducer and activator of transcription 3; UC, ulcerative colitis.

As shown in (Fig. 6A,B), administration of AA significantly increased colonic levels of S1P and its receptor; S1PR1, compared to control rats. Treatment with SLZ and SLZ/Biotin combination therapy significantly reduced colonic levels of S1P by 1.6-folds and 1.9-folds, respectively as compared with UC rats. In addition, SLZ and SLZ/Biotin combination therapy significantly reduced colonic levels of S1PR1 by 1.5- and 1.8-folds, respectively as compared with UC rats. However, biotin-treated rats showed non-significant reduction in colonic levels of S1P and S1PR1 when compared to UC rats.

### Effect of SLZ and biotin on IL-23, STAT3, P-STAT3 colonic levels

As shown in (Fig. 6C–E), AA-induced UC group revealed significant increase in colonic levels of IL-23, STAT3 and P-STAT3, compared to control group. All treatment groups revealed significant attenuation of IL-23, STAT3 and P-STAT3 colonic levels, compared to AA-induced UC group. Moreover, SLZ/Biotin combination therapy group revealed significant reduction in colonic levels of IL-23, STAT3 and P-STAT3 by 2-, 1.6- and 1.7-folds, respectively compared to SLZ-treated group.

### Effect of SLZ and biotin on colonic P-STAT3/STAT3 ratio

The colon of AA-induced UC rats showed marked elevation of P-STAT3/STAT3 ratio by 1.2-folds, compared with control rats. Rats treated with biotin either alone or in combination with SLZ revealed a significant reduction of colonic P-STAT3/STAT3 ratio, by 1.2- and 1.4-, respectively, when compared to AA-induced UC rats. Furthermore, the administration of SLZ/Biotin combination therapy showed a marked reduction in colonic P-STAT3/STAT3 ratio by 1.3-folds, compared to SLZ-treated group (Fig. 6F).

### Effect of SLZ and biotin on colonic NF-κB protein expression

**Fig. 7:**
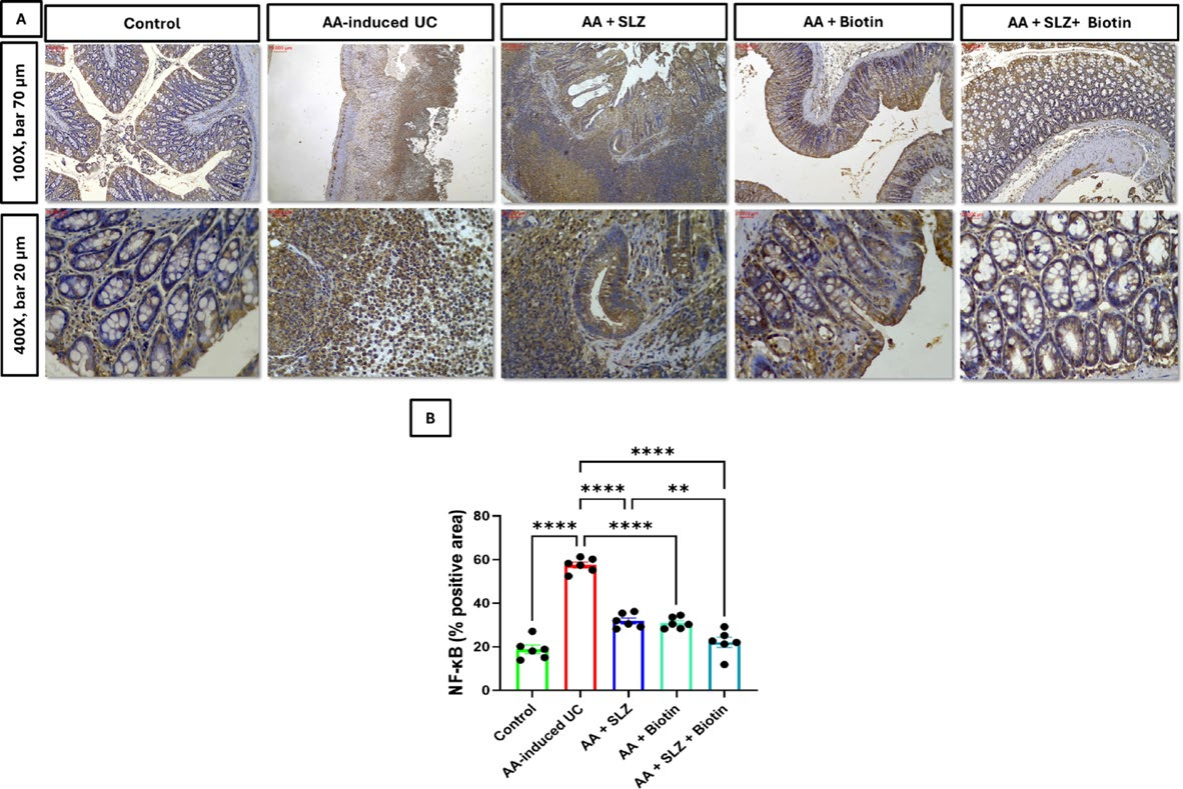
Effect of SLZ and biotin on colonic NF-κB protein expression in AA-induced UC in rats. (**A**) Representative images of NF-κB IHC staining of colon tissues. Upper panel (100X, bar 70 μm) and lower panel (400X, bar 20 μm). (**B**) The % positive area of colon sections stained with anti-NF-κB antibodies. Values are expressed as mean ± SEM. (n = 6). **p < 0.01, ****p < 0.0001. AA, acetic acid; IHC, immunohistochemical; NF-κB, nuclear factor kappa B; SLZ, sulfasalazine; UC, ulcerative colitis.

The AA-induced UC rats revealed significant elevation of percentage area in immunostained colonic tissues with NF-κB by 3-folds, compared to control rats. Groups treated with SLZ, biotin and SLZ/Biotin combination therapy revealed significant reduction in colonic NF-κB protein expression by 1.8-, 1.9- and 2.6-folds, respectively, compared to AA-induced UC group. Moreover, SLZ/Biotin combination therapy group revealed significant reduction in colonic NF-κB protein expression by 1.4-folds, compared to SLZ-treated group (Fig. 7).

### Effect of SLZ and biotin on colonic COX-2 protein expression

**Fig. 8:**
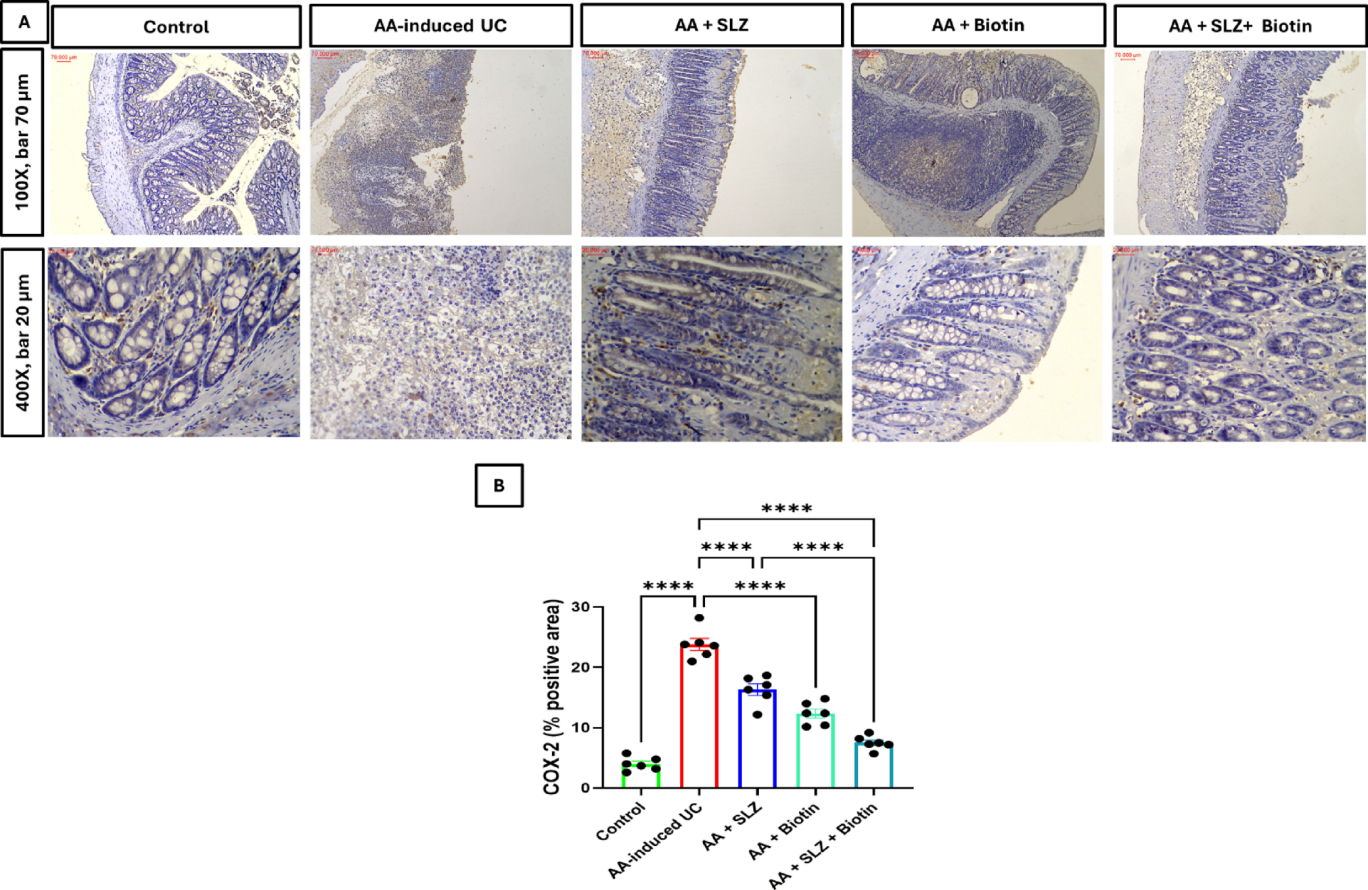
Effect of SLZ and biotin on colonic COX-2 protein expression in AA-induced UC in rats. (**A**) Representative images of COX-2 IHC staining of colon tissues. Upper panel (100X, bar 70 μm) and lower panel (400X, bar 20 μm). (**B**) The % positive area of colon sections stained with anti-COX-2 antibodies. Values are expressed as mean ± SEM. (n = 6). ****p < 0.0001. AA, acetic acid; COX-2, cyclooxygenase-2; IHC, immunohistochemical; SLZ, sulfasalazine; UC, ulcerative colitis.

Investigation of immunostained colon sections from AA-induced UC group showed a significant increment in COX-2 protein expression by 5.9-folds, compared to control group. Groups treated with SLZ, Biotin and SLZ/Biotin combination therapy showed significant lowering in colonic COX-2 protein expression by 1.5-, 1.9- and 3.2-folds, respectively, compared to AA-induced UC group. Moreover, SLZ/Biotin combination therapy group showed significant decrease in colonic COX-2 protein expression by 2.2-folds, compared to SLZ-treated group (Fig. 8).

## Discussion

UC is a local immunological response accompanied by diarrhea, rectal bleeding, loss of weight, as well as colonic macroscopic changes involving ulceration and edema. The shortage of a definitive medication for UC, the virulent prognosis, and deleterious impacts of treatments made it challenge to find novel therapeutic potential agents in order to evade former appearance and prognosis of the disease^31,32^. Thus, the present study assesses the effect of biotin in combination with SLZ against AA-induced UC in rats.

In the current study, the induction of UC in rats was done by intra-rectal AA instillation owing to proton release which promotes acidification of intracellular epithelium and causes massive epithelial injuries. UC induction was manifested by a significant increment in colon mass index, weight/length ratio, macroscopic score, DAI, serum CRP level and LDH activity in addition to the histopathological examination that showed mucosal erosions, ulceration, atrophy, inflammation and hemorrhage in the mucosal and submucosal layers. These observations are in line with former findings^33,34^. Interestingly, SLZ/Biotin combination notably reversed AA-evoked UC and enhanced biochemical markers in addition to restoration of the histopathological structure.

UC is characterized by altered trafficking of immune cells leading to T lymphocytes migration and accumulation in the gut that causes inflammation, together with local immune cells like macrophages, dendritic cells and innate lymphoid cells^35^. A crucial regulator of lymphocyte migration is the S1P/S1PR1 axis. Subsequently, S1P/S1PR1 axis targeting may display a substantial curative strategy for mitigating inflammation in UC. S1P is a bioactive sphingolipid metabolite that exerts different biological and immunologic effects. Moreover, S1P plays important roles in the pathogenesis of colitis. Increased concentration of S1P, detected at the inflammation sites, promote inflammatory signaling, immune cells engagement, and further releasing of other pro-inflammatory agents^36^. High levels of S1P expression accompanied with an elevation in S1PR1 concentration in the inflamed area. S1PR1 is one of G protein-coupled receptors that mediates S1P bioactivity and has an important role in lymphocyte trafficking and inflammation^37^. Previous studies revealed elevation of S1P production and S1PR1 expression after induction of colitis in mice, in consistent with our results^38,39^. Contrariwise, administration of SLZ/Biotin combination therapy attenuated S1P and S1PR1 colonic levels in AA rats. As far as we are aware, this is the first study presented that SLZ/Biotin combination can ameliorate UC via alteration of S1P/S1PR1 signaling pathway.

S1P and its binding to S1PR1 leads to activation of NF-κB and STAT3 signaling pathways^40^. The transcription factor NF-κB is the major regulator of immune response and its activation linked to inflammatory disorders like UC. In the intestinal cells, activation of NF-κB results in formation of several pro-inflammatory cytokines, COX-2 enzyme, oxidative and nitrosative stress which involved in the pathophysiology and progression of UC. Interaction of these factors together leads to overstated inflammation reactions which in turn cause colonic injury in UC^41–43^. Surprisingly, suppression of STAT3 activation is linked to inhibition of NF-κB activation^44^. NF-kB also regulates the expression of IL-23^45^. The IL-23 axis is an emerging treatment target for IBD, including UC^46^. IL-23-driven inflammation is related to the action of T helper type 17. It is connected to the emergence of inflammation and autoimmunity. The binding of IL-23 to its receptor triggers certain intracellular signals, such as STAT3, which controls the transcription of genes encoding many cytokines^47,48^. In this work, UC induction leads to an increase in NF-κB activity and the production of IL-23 in colon tissue which is in accordance with a former study^43^.

Moreover, phosphorylation of STAT3 and its translocation to the nucleus contributes in the regulation of cell migration, differentiation, proliferation and transcription of inflammatory target genes^49,50^. P-STAT3 elevated level is intimately related to inflammation and creation of histological lesions. Furthermore, P-STAT3 activates S1P-S1PR1 signaling pathway and upregulates S1PR1 expression, which in turn preserves STAT3 in its activated state^51,52^. One of the downstream effects of activated STAT3 is increasing COX-2 expression in inflamed tissues^53^. COX-2 is regarded as an essential inflammatory mediator in UC prognosis. The COX-2 role in mediating the barrier dysfunction that participates in colonic inflammation is attributed to regulating prostaglandins production in inflamed mucosa^54,55^. In accordance with this, a significant increase in colonic P-STAT3/STAT3 ratio and a subsequent elevation in COX-2 in colon samples isolated from AA-induced UC group were observed in our study that comes in agreement with previous studies^43,53^.

Treatment with biotin alone and in combination with SLZ reduced colonic P-STAT3/STAT3 ratio, NF-κB and IL-23 levels which consequently decreased COX-2 production. This anti-inflammatory ability of biotin was stated in other previous studies^56,57^. Furthermore, SLZ/Biotin suppressed elevated levels of CRP in UC rats that is a sign of ongoing inflammation and tissue damage.

Oxidative stress is a key component in the pathophysiology of UC. It is an imbalance between pro- and antioxidants that plays a critical role in intestinal inflammation and mucosal tissue damage in colitis. Lipids, DNA and proteins are among the cellular macromolecules that are vulnerable to cellular oxidative damage via attacking by reactive oxygen/nitrogen species and free radicals. AA instillation in colon leads to an elevation in the levels of reactive oxygen species as evidenced by elevated MDA, which represents the lipid peroxidation level in the cells causing an intensive exhaustion of antioxidants defenses in cells like depletion of GSH that impedes its pivotal role in free radicals scavenging^34^. Moreover, UC rats showed a marked increment in NO concentration. NO is a nitrosative stress factor and an indicator of inflammation that has been involved in the etiology of several illnesses. Although NO alone cannot directly damage DNA, it can interact with superoxide and oxygen to generate very reactive compounds: peroxynitrite and dinitrogen trioxide, respectively which in turn cause cytotoxic tissue damage and exaggerate the inflammation^58,59^. However, SLZ/Biotin treatment counteracted oxidative stress as indicated by a marked reduction in MDA and NO concentrations besides a substantial elevation in GSH concentration. These results further potentiate the anti-inflammatory effects of the SLZ/Biotin combination.

**Fig. 9:**
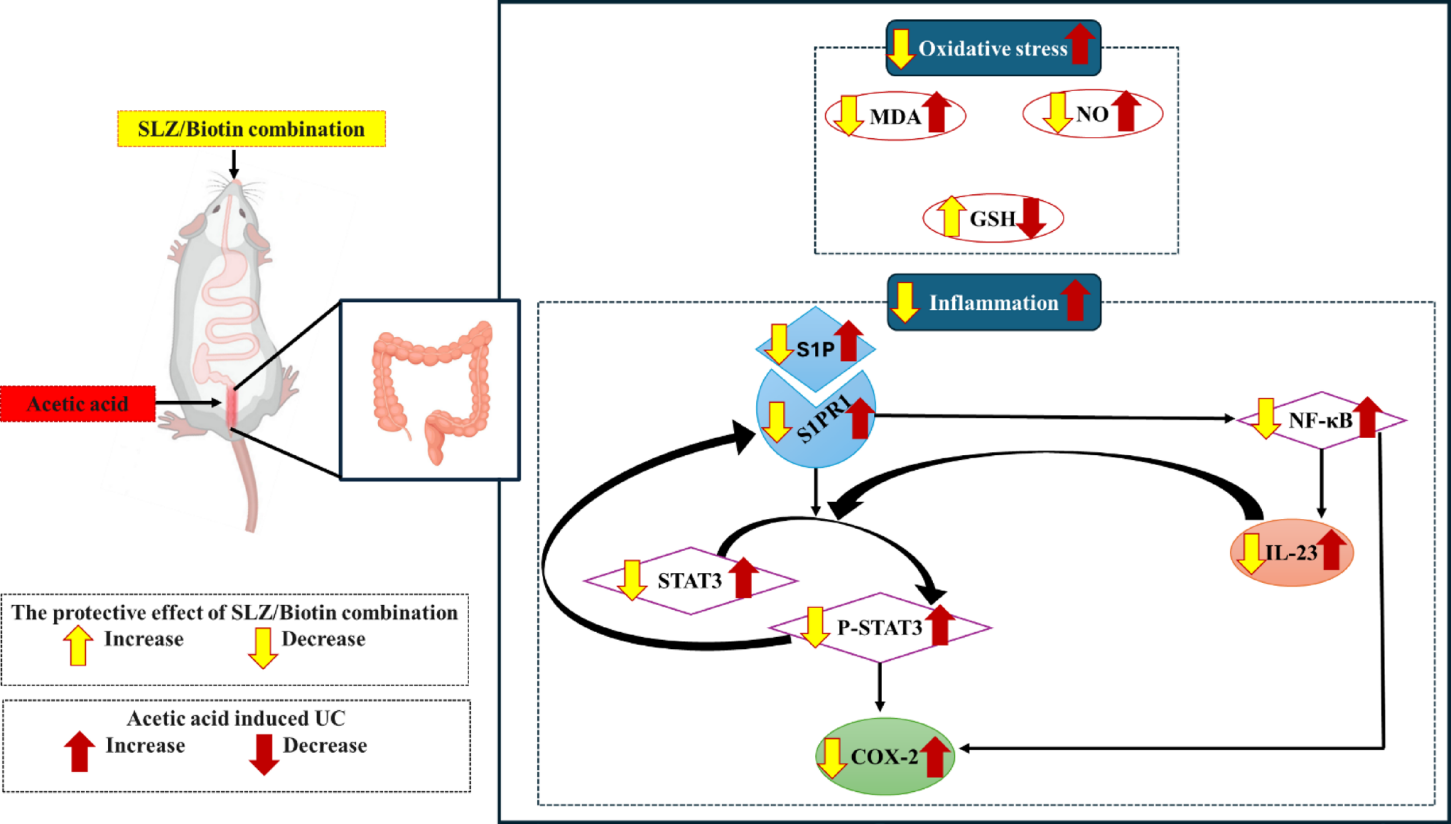
Schematic presentation summarizing the proposed mechanisms underlying SLZ/Biotin combination in alleviating AA-induced UC. SLZ/Biotin combination downregulated S1P and S1PR1 which in turn decreased NF-kB and STAT3 with subsequent decrease in COX-2 levels in colonic tissues. In context, the colonic level of IL-23, the downstream target of NF-kB, was suppressed, which in turn decreased STAT3 signaling leading to amelioration of inflammatory response. Moreover, SLZ/Biotin combination restored redox balance in colonic tissues. AA, acetic acid; COX-2, cyclooxygenase-2; CRP, C-reactive protein; GSH, glutathione; IL-23, interleukin-23; LDH, lactate dehydrogenase; MDA, malondialdehyde; NF-κB, nuclear factor kappa B; NO, nitric oxide; S1P, sphingosine-1 phosphate; S1PR1, sphingosine-1 phosphate receptor 1; SLZ, sulfasalazine; STAT3, signal transducer and activator of transcription 3; UC, ulcerative colitis.

Collectively, the modulatory effect of SLZ/Biotin against AA-induced UC in rats could be attributed to inhibiting oxidative stress and inflammatory response via targeting S1P/S1PR1/NF-kB/ IL-23/ STAT3 signaling pathway with subsequent inhibition of COX-2. Biotin as an add-on strategy to SLZ may be useful for ameliorating UC. Figure 9 summarizes the proposed mechanisms underlying SLZ/Biotin combination in alleviating AA-induced UC.

Among the chemically- induced UC, AA-induced colitis is the most common and easily inducible rat model resembling pathogenesis, histopathology, and some immunology of human disease^60^. However, it lacks some of the complex immune dysregulation seen in human UC and this is considered the limitation of our study.

## Data Availability

All data generated or analyzed during this study are included in this published article.

## Abbreviations

AA: Acetic acid
CMC: Carboxymethyl cellulose
COX-2: Cyclooxygenase-2
CRP: C-reactive protein
DAI: Disease activity index
GSH: Glutathione
H&E: Hematoxylin and eosin
IBD: Inflammatory bowel disease
IHC: Immunohistochemical
IL-23: Interleukin-23
LDH: Lactate dehydrogenase
MDA: Malondialdehyde
NF-κB: Nuclear factor kappa B
NO: Nitric oxide
S1PR1: Sphingosine-1 phosphate receptor 1
SLZ: Sulfasalazine
STAT3: Signal transducer and activator of transcription 3
UC: Ulcerative colitis
